# Development of a Food-Based Diet Quality Scale for Brazilian Schoolchildren Using Item Response Theory

**DOI:** 10.3390/nu13093175

**Published:** 2021-09-12

**Authors:** Simone de C. Giacomelli, Maria Alice A. de Assis, Dalton F. de Andrade, Jeovani Schmitt, Patrícia de F. Hinnig, Adriano F. Borgatto, Raquel Engel, Francilene G. K. Vieira, Giovanna M. R. Fiates, Patricia F. Di Pietro

**Affiliations:** 1 Post Graduate Program in Nutrition, Health Sciences Center, Federal University of Santa Catarina, Campus Trindade, Florianopolis 88040-900, SC, Brazil; scgiacomelli@gmail.com (S.d.C.G.); malicedeassis@gmail.com (M.A.A.d.A.); patricia.hinnig@ufsc.br (P.d.F.H.); frankunradi@gmail.com (F.G.K.V.); gifiates@gmail.com (G.M.R.F.); 2 Informatics and Statistics Department, Technological Center, Federal University of Santa Catarina, Campus Trindade, Florianopolis 88040-900, SC, Brazil; dalton.andrade@ufsc.br (D.F.d.A.); adriano.borgatto@ufsc.br (A.F.B.); 3 Department of Education, Catarinense Federal Institute of Education, Science and Technology, Rua Bernardino José de Oliveira, 81, Blumenau 89070-270, SC, Brazil; jeovani.schmitt@gmail.com; 4 Department of Nutrition, Avantis University Centre, Av Marginal Leste 3600, Balneario Camboriu 88339-125, SC, Brazil; raquelengel_nutri@hotmail.com

**Keywords:** psychometrics, diet, food consumption, child, surveys and questionnaires

## Abstract

Item response theory (IRT) is a psychometric method that provides probabilistic model-based measurements. Its use is relatively recent in the assessment of food consumption, especially through dietary assessment tools. This study aims (1) to develop a food-based diet quality scale for Brazilian schoolchildren using IRT, and (2) to apply the scale to a representative sample of schoolchildren from a Southern Brazilian city. The scale was developed with daily consumption frequency of foods from 835 students who completed the Food Intake and Physical Activity of Schoolchildren questionnaire. Questionnaire foods were grouped into 10 items according to their nutritional similarities and were evaluated by full-information factor analysis that indicated a dominant factor explaining 28% of the variance. Psychometric item analysis was performed using Samejima’s model. The scale covered all levels of diet quality, from “very poor” (scores < 95) to “very good” (scores ≥ 130). Children who had higher diet quality scores consumed beans, meat, fish, eggs, fruits, vegetables, dairy products, and water more frequently, while reducing the consumption of ultraprocessed sugary foods, ultraprocessed savoury snacks and sausages, and sugary drinks. Of 6323 children, an average of less than 10% consumed the highest diet quality scores (good or very good diet quality) and about 60% of children consumed low diet quality scores. The scale can be applied to other schoolchildren with the same measure precision.

## 1. Introduction

Dietary indices evaluate diet quality, usually based on updated food-based dietary guidelines [1,2,3]. Short food questionnaires (SFQ) are emerging as appealing brief assessment methods to collect data of specific dietary components (e.g., healthy food markers such as fruit, vegetables, grains, dairy products, and meat, and unhealthy food markers such as savoury snacks, processed meats, and sugary drinks) from which to derive a diet quality score [1,2,3]. They are shorter and less detailed than 24 h food recalls, food records, or food frequency questionnaires (FFQ) as they do not quantify total energy intake, rather reporting frequency or number of servings of foods ingested [1,2,3]. They are useful for monitoring and surveillance, as indicators of overall diet quality when individual nutrient and energy assessment are not the main focus [1,2,3].

Traditionally, the construction, scoring, refinement, calibration, and validation of FFQ and SFQ, as well as development of diet quality scales are based on classical test theory (CTT) [3,4]. A measure’s properties under CTT may vary across different populations, requiring new evaluations such as reliability and validity whenever the scale is used in a new population [5,6].

An alternative model-based theory is item response theory (IRT), which expresses the probability of a particular response to a questionnaire item as a function of the quantitative attribute (latent trait) of the person and the items’ parameters [5,6,7,8].

By modelling the relationship of individual items to the construct being measured, IRT provides a richer description of the performance of each item, which is useful during the development of diet quality scales to ensure that the best items are selected. Information functions provided through IRT describe how precision may vary across different levels of the latent trait at the item or scale level [7,8]. In contrast, using CTT, a single estimate (e.g., Cronbach’s α value) is used to describe a measure’s reliability [5,9]. When key assumptions are met, IRT offers the property of item invariance, the latent trait estimates are independent of the specific item set, and the item parameters are independent of the sample of respondents [5,7]. In addition, both items and individuals can be placed on a common metric along the latent trait continuum [7,9].

Examples of IRT models applied in nutrition research include the evaluation of psychometric properties of an item bank for assessing how food parenting practices influence child dietary behaviours [10], the development and refinement of scales to measure health motivation influencing food choices in European adolescents [11], and behaviours of adolescents and adults related to the recommendations of the 2014 Dietary Guidelines for the Brazilian Population [12].

However, IRT models have been scarcely applied to develop and refine dietary assessment methods such as SFQ [6], or indexes of overall diet quality.

The Web-CAAFE (Food Intake and Physical Activity of Schoolchildren) is a validated SFQ designed for 7–10-year-old Brazilian schoolchildren to provide information on obesity-risk-related dietary behaviours [13,14,15,16,17,18]. It may be a suitable tool to which an index can be applied; however, new psychometric approaches to develop such measures call for better analytical methods beyond what CTT can provide.

The Web-CAFFE was developed and tested for use with schoolchildren, considering the cognitive development of 7–10-year-old schoolchildren, and underwent validity and reproducibility studies in two Brazilian cities [14,15,16]. The Web-CAAFE uses illustrations for foods and beverages in each eating event, appropriate for children. Food intake, portion sizes and cognitive processes differ between adults and children, and the use of adult food consumption instruments by the latter could overestimate food consumption, discouraging its use in this age group [2]. The aims of this study were: (1) to develop a food-based diet quality scale for Brazilian schoolchildren (Escala de Qualidade da Dieta de Escolares, EQUADE) using IRT; and (2) to apply the scale in a dataset of reported food intake by a representative sample of schoolchildren from public schools in a Southern Brazilian city.

## 2. Materials and Methods

**Figure 1:**
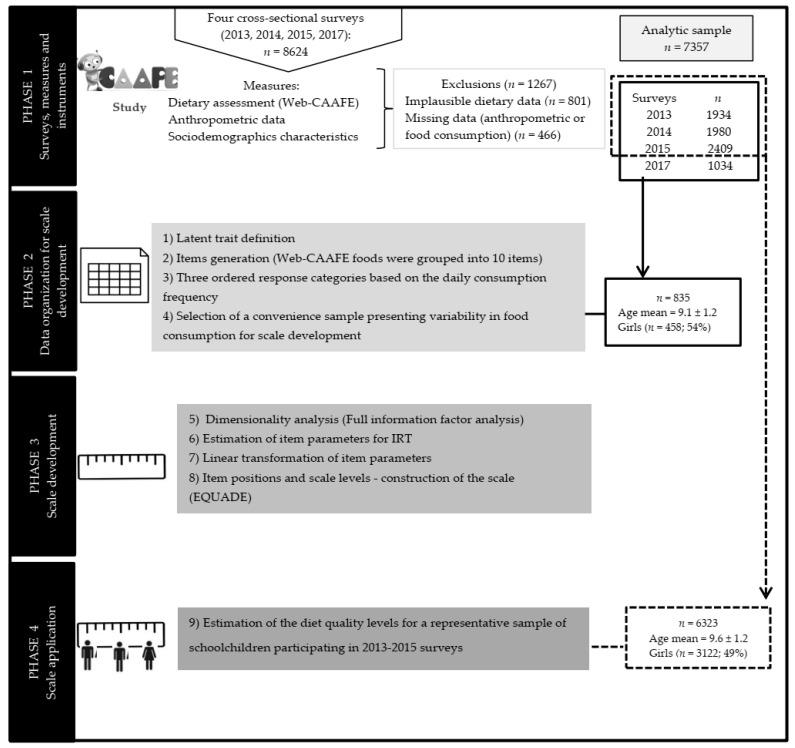
Flowchart of the four phases and nine steps of scale development and application (CAAFE, Food consumption and physical activity of schoolchildren; EQUADE, Schoolchildren’s Diet Quality Scale; IRT, Item Response Theory).

This is a psychometric study using IRT analysis [7,8,9]. The study has four phases and nine steps. A flowchart of the study design and participant selection for the analyses is presented in Figure 1.

### 2.1. PHASE 1-Surveys, Measures and Instruments

The Web-CAAFE study was designed for nutritional surveillance comprising cross-sectional surveys applied periodically at the school setting in order to obtain repeated measures of food intake, physical activity, sedentary behaviour and weight status of schoolchildren attending 2nd to 5th grades of public elementary schools (7–12 years old) in Florianopolis (south of Brazil). Data collections were performed from August to November in 2013, 2014, 2015, and 2017.

The description of the Web-CAAFE study design and sampling procedure has been published previously [17,18]. In brief, for the 2013–2015 surveys, primary sampling units were eligible classrooms (2nd to 5th grades) which were randomly selected from the complete list of schools with computer rooms (34 out of 37 schools with 6227 students enrolled in 2013; 34 out of 36 schools with 6500 students in 2014; 35 out of 36 schools with 7104 students in 2015) [17,19]. For the 2017 survey, all public schools serving the 2nd to 5th grades with a computer room were considered eligible (19 schools), representing the five geographical regions of the city. Nine schools were randomly selected with 2333 students enrolled [18]. All students from 2nd to 5th grades were invited to participate. The Web-CAAFE study (2013–2017) was carried out in a sample of 8624 schoolchildren. A total of 801 children with implausible dietary data (i.e., consumption of less than four food items per day (*n* = 267) or values outside the mean + 3 standard deviation (*n* = 534)) and 466 children with missing data (for body weight and height and food consumption) were excluded. The final sample included 7357 schoolchildren (1934 in 2013; 1980 in 2014; 2409 in 2015; and 1034 in 2017).

This study was conducted according to the guidelines set out in the Code of Ethics of the World Medical Association (Declaration of Helsinki) and approved by the Ethical Committee of the Federal University of Santa Catarina. All parents or guardians received a written consent form with explanations about the procedures adopted in the research (anthropometry and questionnaire on food consumption and physical activity) and were asked to sign the terms, agreeing or not with their child’s participation. The children’s assent was obtained for participants in all four surveys.

#### 2.1.1. Weight Status and Family Income

Weight and height measurements were performed according to standard procedures [20], and taken on lightly dressed barefoot children on the same day that schoolchildren answered the Web-CAAFE. Body weight was measured to the nearest 0.1 kg with a portable digital scale (Marte^®^, model PP, São Paulo, Brazil). Height was measured with a portable stadiometer to the nearest 1 mm (AlturExata^®^). The schoolchildren’s weight status was classified according to the BMI cut-off points for age and sex of the International Obesity Task Force [21] as non-overweight (including thinness and normal weight) and overweight (including obesity).

Average census sector income of the school location area was available from the Brazilian Institute of Geography and Statistics [22] and used as a proxy for the family income because the family residential address determined the school a child was assigned to attend. These values were treated at the individual level.

#### 2.1.2. Web-CAAFE Questionnaire

The Web-CAAFE is an SFQ designed for 7–10-year-old children, structured in three sections: registration, diet, and physical activity [13]. Web-CAAFE was tested for both reproducibility and validity [14,15,16]. Validity tests of the food consumption section, using direct observation at school meals as the reference method, showed 43% matches, 29% intrusions, and 28% omissions [14], placing this questionnaire’s accuracy close to that of other similar instruments [23,24]. The demo version of the questionnaire is available at: CAAFE questionnaire. Available online: http://caafe.ufsc.br/portal/10/detalhes (accessed on 21 August 2021).

Web-CAAFE assesses food consumption of the previous day and was developed to investigate the consumption frequency of specific foods (not nutrients) [17,18]. The food selection displayed as images/icons in the diet section took into account their frequency of intake in this age group as reported previously by 180 schoolchildren in 7-day food diaries, the foods presented in school menus [13], as well as the foods recommended in the Dietary Guidelines for the Brazilian Population (DGBP) [25,26].

The 2008 and 2014 editions of the DGBP differ in their dietary recommendations. The 2008 edition [25] emphasizes the intake of foods corresponding to eight food groups, recommending the number of servings/portions per day: cereals, tubers, roots, and derivatives (6 portions/day); beans (1); fruits (at least 3); vegetables and greens (at least 3); milk and dairy products (3); meat and eggs (1); at most one portion for oils, fats and oilseeds; at most one portion for sugars and sweets. It also recommends the restriction or eventual consumption of foods with excess of fats and salt, soft drinks, industrialized drinks, sweets and confectionery products [25]. The 2014 edition [26] does not quantify portions of food groups that should be eaten on a daily basis and covers the following food groups: beans; cereals; roots and tubers; vegetables and greens; fruits; chestnuts and walnuts; milk and cheese; meat and eggs; and water. These guidelines presented a food classification system categorized according to their nature, extent and purpose of processing into unprocessed or minimally processed foods, processed culinary ingredients, processed foods, and ultra-processed foods [26].

#### 2.1.3. Dietary Assessment

Children completed the instrument under researchers’ supervision in the school computer room. The children were then instructed to indicate which foods they ate at each meal of the previous day.

The diet section is divided into six daily meals ordered chronologically: breakfast, mid-morning snack, lunch, afternoon snack, dinner, and evening snack. For each meal, icons of 31 foods, beverages, or food groups are displayed on the computer screen: rice, corn/potatoes/mashed potatoes, cassava flour, pasta, bread/biscuits, cheese bread, cakes, cooked beans, vegetables, leafy greens, vegetable soup, fruits/fruit salad, milk, coffee with milk, cheese, yoghurt, beef/poultry, fish/seafood, eggs (boiled, fried, or omelette), sweets, sandwich cookies, breakfast cereal, sodas, fruit juices, chocolate milk, instant noodles, French fries, sausages, chips, pizza/hamburgers/hot dogs, and water.

The frequency of food intake was defined as the number of times per day, ranging from 0 to 186 (31 food/beverages × 6 meals), assuming that only one serving was consumed on each occasion. As the Web-CAAFE does not provide data on quantities (portion size or grams) of foods, we were not able to apply exclusion criterion based on energy misreporters to exclude children with implausible consumption. Thus, assuming a Poisson distribution for the frequency of food consumption [17], so that the standard deviation (SD) equalled the square root of the mean, we considered implausible dietary data, such as reporting fewer than four food items per day, or values outside the mean + 3 SD.

Food intake was measured once for every child and the day at which the questionnaire was assessed differed between children. This strategy was used in order to describe the daily variability of dietary intake over school days (Monday to Thursday) and non-school days (Sunday and holidays), allowing for analysis at the group level. As the Web-CAAFE was applied in the school setting and there was no school on Saturdays and Sundays, it was not possible to obtain data representing food intake for Fridays and Saturdays.

### 2.2. PHASE 2-Data Organization for Scale Development

#### 2.2.1. Step 1—Latent Trait Definition

The “diet quality” was defined as the intake frequency of healthy and unhealthy food markers according to the DGBP [25,26]. As with most dietary guidelines, the diet quality was intended to relate to public health recommendations and is similar to those used in other countries [27,28,29,30].

#### 2.2.2. Step 2—Items Generation

Web-CAAFE foods and beverages were grouped into 10 items according to their nutritional similarities based on the food groups of the DGBP [25,26]. Table 1 presents the seven items classified as healthy foods (items 1–6 and 10), and the three food groups considered unhealthy items (items 7–9).

#### 2.2.3. Step 3—Response Categories

Three ordered response categories were defined for each of the 10 items, based on the daily consumption frequency of healthy and unhealthy foods (Table 1). Category 0 (lower) corresponds to the non-consumption of healthy food items (cereals, pasta, breads, rice, roots and tubers; beans; vegetables and leafy greens; fruits; dairy products; meat, fish, and eggs; water) and/or to the consumption of unhealthy food items ≥ 2 times/day (ultraprocessed sugary foods; sugary drinks; ultraprocessed savoury snacks and sausages). Category 1 (intermediate) includes the consumption of healthy food items at a frequency below or above the DGBP recommendations and the consumption of unhealthy food items 1 time/day. Category 2 (higher) corresponds to the consumption of healthy and unhealthy food items at the recommended frequency according to the DGBP [25,26]. The response categories were numbered in ascending order in line with the cumulative feature of the latent trait. Thus, category 0 (lower) identifies the answer option whose consumption suggests a worse diet quality, while category 2 (higher) suggests a better diet quality.

#### 2.2.4. Step 4—Selection of a Convenience Sample Presenting Variability in Food Consumption

A convenience sample of 7–12-year-old schoolchildren (835 out of 7357 schoolchildren; 54.8% girls and 45.2% boys; mean age ± SD, 9.1 ± 1.2; 27.3% overweight (including obesity); mean family income (R$) ± SD, 2005 ± 969) was selected to estimate item parameters and construct the scale. According to the invariance principle of IRT, item parameters (discrimination and difficulty) are independent of the population of respondents. Thus, the sample selected for the construction of the scale must be representative of the latent trait (quality of the diet) and not of the population [7,8]. The convenience sample was selected from the entire sample assuring representativeness of respondents in all levels of the latent trait, i.e., responses in each of the categories of every item [7,8]. Thus, schoolchildren who consumed food according to response categories 0, 1, and 2 of the ten generated items were selected.

### 2.3. PHASE 3-Scale Development

#### 2.3.1. Step 5—Dimensionality

The unidimensionality assumption was analysed by full-information factor analysis, an appropriate approach to treat a set of items using ordinal response categories [31]. We observed the variance explained by the dominant factor (acceptable 20% or more of the total variance) [32], the scree plot with eigenvalues >1.0, and the factor loadings of items ≥0.30.

#### 2.3.2. Step 6—Item Parameters

Using the Samejima’s graded response model [33] for ordered response categories, the discrimination (*a_i_*) and the difficulty (*b_i_*) parameters were estimated. The discrimination parameter represents the quality of an item to discriminate respondents with different levels along the latent continuum. The difficulty parameter, also called the location or position parameter, identifies the point on the latent trait scale where there is a 50% probability that a given response category or a higher category is chosen. It represents the threshold between response categories of the item.

Supposing that the categories of an item *i* are arranged from lowest to highest and denoted by *k_i_*, the probability that an individual j chooses a particular category *k_i_* or a higher category of item *i* is given by the equation [33]:

(1) $$P_{i,ki}^{+}\left(\theta_{j}\right)=\frac{1}{1+e^{-a_{i}\left(\theta_{j}-b_{i,ki}\right)}}-\frac{1}{1+e^{-a_{i}\left(\theta_{j}-b_{i,ki+1}\right)}}$$

where *i* = 1, 2, …, *I* (*I* denotes the number of items in the test);

*j* = 1, 2, …, *n* (*n* denotes the total number of respondents);
*k_i_* = 0, 1, …, *m_i_* (*m_i_* denotes the number of categories of the *i*-th item minus 1);
*b_i_*_,_*_ki_* is the difficulty parameter of the *k_i_*-th category of item *i*, with *b_i_*_,1_ ≤ *b_i_*_,2_ ≤ … ≤ *b_i_*_,m_;
*a_i_* is the discrimination parameter of item *i*;
*θ_j_* represents the latent trait of the *j*-th individual, i.e., the individual’s diet quality; and
$P_{i,ki}^{+}\left(\theta_{j}\right)$ is the probability of the *j*-th respondent with a diet quality level of *θ_j_* to be classified in a particular category of the *i*-th diet quality level (*k_i_*) or higher.

Item parameters (*a_i_* and *b_i_*_,*ki*_) were estimated by marginal maximum likelihood and analysed with the corresponding standard error (SE) and item characteristic curves (ICC). The test information function was determined to identify the accuracy of the measurement along the scale [7]. Individual scores were estimated using the weighted likelihood estimation method [34]. IRT analyses were performed using the PSYCH and MIRT packages in R software [35].

#### 2.3.3. Step 7—Linear Transformation of Items Parameters

All item parameters were initially standardised to have a mean of zero and a standard deviation of one (metric 0,1); however, a linear transformation was performed to obtain a mean of 100 and a standard deviation of 10 (metric 100,10), respecting the existing linear relations between points. Linear transformation was used to avoid negative or decimal numbers, facilitating scale utilisation and interpretation [7].

#### 2.3.4. Step 8—Item Positions and Scale Levels

The construction of the EQUADE was performed by placing the response categories of each item at a cumulative probability point of 0.60; i.e., at the point where there was ≥ 60% probability that students of a certain level of diet quality would consume a food item according to a specific item response category (or a higher category).

Each level was formed by the items placed within the established interval. The cut-off points for each level considered the gain in diet quality with food intake characteristics. This step was performed by two authors (SCG and PFDP) using Microsoft Excel^®^ version 2010. Scale levels and their descriptions were reviewed by six nutrition experts.

### 2.4. PHASE 4, Step 9—Scale Application

The EQUADE was applied in a representative sample of elementary schoolchildren to estimate the level of their diet quality using the complex sampling plan created for the data selected from 2013 to 2015 (*n*, 6323). The sampling plan was stratified in two levels, the strata being the combination of Year and School; level 1 was the class and level 2 the student within the class. The Rao–Scott chi-square test was used to analyse differences between survey years and between diet quality and income. Differences between diet quality and income in tertiles were also performed using the same test. Analyses were performed using the survey package of R software [36].

## 3. Results

The assumption of unidimensionality was supported as the eigenvalues from the full information factor analysis on the set of items showed the presence of a ‘dominant’ factor (i.e., the first factor accounted for 28% of the variance) and the 10 items presented factor loadings >0.3, indicating that they are closely related to the latent trait (Supplementary Table S1 and Supplementary Figure S1).

The estimated item discrimination and difficulty parameters are presented in Table 2. Item discrimination parameters ranged from 0.68 to 1.85, indicating that all items had a satisfactory discriminating power. The item ‘Cereals, pasta, breads, roots, and tubers’ had the lowest discrimination parameter (*a_i_* = 0.68) and ‘Water’ the highest (*a_i_* = 1.85). The size of standard error agreed with those of the corresponding item parameters, indicating that they were well estimated.

The item “Cereals, pasta, breads, roots, and tubers” had the lowest difficulty parameter in the intermediate category (*b*_1_ = −3.34), indicating that schoolchildren easily consumed these foods one to four or ≥ seven times/day. In the higher category, ‘Meat, fish, and eggs’ had the lowest difficulty parameter (*b*_2_ = −0.35), suggesting that consumption one to two times/day was relatively easy.

“Vegetables and leafy greens” had the highest difficulty value in the intermediate category (*b*_1_ = 0.23). In the higher category, ‘Fruits’ (*b*_2_ = 3.36) and ‘Vegetables and leafy greens’ (*b*_2_ = 3.13) presented the highest difficulty parameters, suggesting that its consumption at least three times/day was the most difficult.

**Figure 2:**
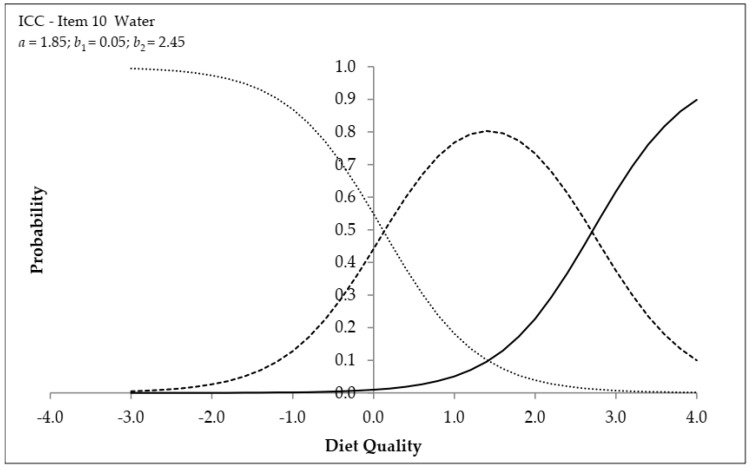
Item characteristic curve for the item ‘Water’. The dotted line indicates the lower response category (0 times a day), the dashed line indicates the intermediate category (one to four times a day), and the solid line indicates the higher category (5 to 6 times a day).

Figure 2 presents the ICC for ‘Water’. Schoolchildren with a diet quality of up to 0.05 are more likely to not drink water (lower category). Schoolchildren with a diet quality between 0.05 and 2.45 are more likely to drink water one to four times/day (intermediate category), and those with a diet quality above 2.45 are more likely to drink water five to six times/day (higher category). The inflection point of the lower and higher category curves are *b*_1_ of 0.05 and *b*_2_ of 2.45, respectively. The ICC of other items are presented in Supplementary Figures S2 and S3.

**Figure 3:**
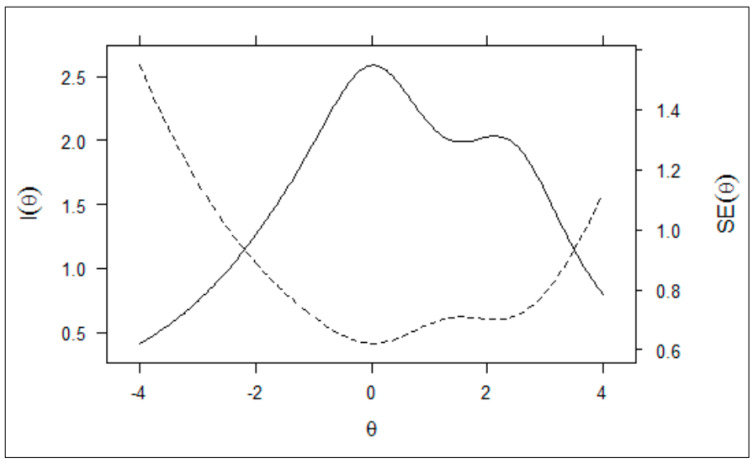
Test information (solid line) and standard error of measurement (dashed line) curves of the Schoolchildren’s Diet Quality Scale.

Figure 3 shows the test information curve (solid line), which represents the sum of information for all items on the scale, and the standard error of measurement curve (dashed line). The plot indicates the region in the scale where the accuracy of diet quality measurement is highest. From this information, it is possible to determine the scale range that is most suitable for evaluating diet quality. The instrument covers the entire latent trait, indicating that it is particularly suitable for measuring diet quality scores ranging from −2.0 to 3.5. However, medium scores on the scale are more informative than those in low and high levels, because the higher the value of the information and the lower the standard error of measurement, the greater the accuracy of the diet quality estimates.

Table 3 shows the position of items on a scale (metric 100, 10). Scale levels were defined on the basis of item positions allowing interpreting schoolchildren’s scores at each level. Five progressive and cumulative levels of diet quality were established, varying from very poor (level 1) to very good (level 5).

Table 4 shows the description of each diet quality level based on the five levels of scale scores. As levels progress, there is a gain in diet quality according to the characteristics of food consumption. The first level (*θ* < 95), ‘very poor’ diet quality, reflects a poorly varied diet and rich in unhealthy foods. The highest level (*θ* ≥ 130), ‘very good’ diet quality, indicates a varied diet, including the non-consumption of unhealthy foods and the consumption of the healthy foods at a recommended frequency (higher category).

The descriptive characteristics of schoolchildren that the EQUADE was applied are presented in Supplementary Table S2. Between the surveys years the mean age varied from 9.5 to 9.7 years, the proportion of boys and girls was similar and the prevalence of overweight (including obesity) varied from 23.8% to 27.7%.

The estimates of schoolchildren according to diet quality levels based on three Web-CAAFE survey years (2013–2015) are presented in Table 5. The majority of students were positioned at the level characterized as having a very poor and a poor diet quality. The estimates of students in each scale level did not differ between years. No association was found between diet quality and income (*p* = 0.1221).

## 4. Discussion

The novelty of the present study is the application of a graded response model of IRT to develop a food-based diet quality scale to evaluate how closely schoolchildren’s food patterns align with DGBP [25,26]. A 2020 systematic review [3] of the a priori DQS/index used in children and adolescents identified 128 studies; most of them have used CTT for construction, scoring, and validation, and none of the studies have applied the IRT. IRT does not replace CTT; however, it can be used to increase test quality. For instance, IRT allows determining the standard error of measurement for each respondent and for each item, as well as for each level of the scale, in contrast to CTT, which nearly always assumes the same standard error for all trait levels [9].

The test information curve indicated that the instrument covers the entire range of the latent trait, from ‘very poor’ to ‘very good’ diet quality but is more precise in the range of medium scores. The items had sufficient discriminating power (*a_i_* ≥ 0.7) to measure the latent trait [7,8], meaning that items could be used to discriminate between children with different levels along the latent continuum. ‘Cereals, pasta, breads, roots, and tubers’ presented the discrimination parameter (*a_i_* = 0.68) slightly below the limit for good discriminatory power but was not excluded because of its importance for measuring the latent trait.

The items ‘Meat, fish, and eggs’ and ‘Beans’ had the lowest difficulty parameters, indicating that, even with a low diet quality score, schoolchildren have a higher probability of endorsing a higher response option (one to two times/day). The low difficulty can be explained by the food culture of the Brazilian population, which includes two large meals a day (lunch and dinner) rich in foods belonging to these items [26]. In contrast, ‘Vegetables and leafy greens’ and ‘Fruits’ had the highest difficulty parameter, suggesting that compliance with the recommendation for fruit and vegetable consumption (≥three times/day) was the most difficult. Previous studies reported that schoolchildren have a low intake of fruits and vegetables [37,38].

IRT models aim to estimate the probability that a respondent will provide a particular response to a given item, depending on the respondent’s location on the latent trait continuum [8]. Thus, observing the sequential order of items positioned along the scale, it is possible to identify food consumption behaviours that result in a higher-quality diet. For example, schoolchildren start consuming fruits and vegetables one to two times/day only after reducing the consumption of unhealthy food items to one time/day. Likewise, children start consuming milk and dairy products three times/day and water five to six times/day after they do not consume sugary drinks (Table 3).

Because item parameters and diet quality are located on the same scale (metric) [7,9], it was possible to qualitatively describe each level. Interpretation of the EQUADE allowed identifying food consumption adopted by children and those that need to be stimulated for improvements in diet quality. The first level of diet quality was characterized by a high consumption (≥two times/day) of unhealthy food markers (sweets, sugary drinks, ultraprocessed savoury snacks, and sausages) and added to a low consumption of healthy foods. Diet quality increases as children consume healthy foods markers more frequently, such as beans, meat, fish, eggs, fruits, vegetables, dairy products, and water, while reducing the consumption of unhealthy foods. At the highest level, children do not consume unhealthy foods. As it was possible to produce a meaning for the scores, practitioners can use this tool to assess and monitor the diet quality. In addition, the tool can be used to guide children and their parents about what aspects need to be modified to achieve higher levels of diet quality.

In this study, about 60% of schoolchildren had lower scores (very poor and poor diet quality) and only about 10% had higher scores (good and very good diet quality). Poor diet quality in childhood has been associated with negative health outcomes, including high diastolic blood pressure and precocious puberty [39,40]. These results highlight the need for food and nutrition education interventions aimed at schoolchildren.

IRT analysis allowed us to estimate diet quality based on food consumption behaviour rather than on nutrients. An index that aims to promote health, nutrition education and public policy development should be guided by foods rather than nutrients [4].

The current study has limitations. First, as an SFQ designed for schoolchildren to self-report food consumption, the Web-CAAFE does not assess portion size and does not provide an estimate of total energy, cooking fats and type of foods according to fat content, types of cereal products (e.g., refined or whole grains), types of soft drinks and fruit juices (e.g., regular or diet), or types of cooking methods. The cognitive task required for estimating these details may not be compatible with the perceptual and conceptual capacities of children who have not reached the stage of abstract reasoning at approximately 10–11 years of age [41]. However, the questionnaire is age appropriate and has been developed, piloted, and validated in the study population prior to use. Second, measurement errors, both random and systematic, are present in all self-reported dietary assessment methods, due to factors such as recall error and social desirability bias [42]. In order to minimize reporting errors from self-reports of schoolchildren in the Web-CAAFE, implausible dietary data were not included in the final analytical sample. Third, despite the fact that food consumption based on one-day dietary recall does not necessarily capture the usual intake at the individual level, this method is widely accepted to assess food intake at the population level [42,43]. Finally, item generation took into account the food groups of the DGBP [25,26] recommended on a daily basis, and some foods were categorized into a single group, even with some distinctive nutritional properties. For example, despite fish consumption being considered a healthier option, it was grouped with meat and eggs, because the DGBP [25,26] and other international dietary guidelines [27,28,29,30] recommend fish consumption based on weekly frequency (e.g., two times/week). As the latent trait has a cumulative feature, it would not be feasible to propose daily response categories for fish and another for meat and eggs, because according to the Brazilian culture these foods are consumed in specific meals, at lunch or dinner, and generally are substitutes for each other.

Among the strengths of this study is that we highlight that the EQUADE was constructed based on statistical analyses that sought to solve the limitations of the CTT approach. Item parameters and scores had low standard error, indicating precision of the estimates. Furthermore, the EQUADE is composed of 10 items, comprising healthy and unhealthy food markers, reflecting indicators in alignment with the DGBP. In addition, the interpretation of scale levels provides information for use in diet quality assessment studies.

The scores calculated by the IRT are independent of the respondents’ characteristics, which allows the scale to be applied to other schoolchildren populations with the same measure precision. Even with this statistical basis, researchers who wish to apply the scale must verify if the theoretical understanding of the measured latent trait is the same in the population of interest. If the scale does not evaluate some foods or food groups for the latent trait, IRT allows the inclusion of new items in the scale in future studies.

Further implementation of the EQUADE includes evaluation of the construct validity by assessing its relationships with the intended diet-related health outcomes (e.g., overweight or obesity) to verify if associations are in the expected directions. We also aim to analyse its performance in prospective studies to track diet quality from childhood to adolescence, in order to evaluate its predictive validity against health outcomes.

## 5. Conclusions

IRT analysis allowed developing the EQUADE and to produce a meaning for the scores. Most schoolchildren to whom the scale was applied had low scores in diet quality. Health promotion efforts and nutrition education need to be targeted for schoolchildren. The proposed scale can be applied to other schoolchildren in different settings with the same measure precision, contributing to diet monitoring and to the effective understanding of food consumption measures. Identification of diet quality can guide interventions and public policies in the field of nutrition.

## Figures and Tables

**Table 1:** Ordered response categories for each item according to daily consumption frequency.

Item	Web-CAAFE Foods, Beverages, and Food Groups	Consumption Frequency (Times/Day)		
		Category 0 (Lower)	Category 1 (Intermediate)	Category 2 (Higher)
(1) Cereals, pasta, breads, roots, and tubers	Rice, corn/potatoes/mashed potatoes, pasta, cassava flour, bread/biscuits, cakes, cheese bread	0	1, 2, 3, 4, or ≥7	5 or 6
(2) Beans	Beans	0	≥3	1 or 2
(3) Vegetables and leafy greens	Vegetables, leafy greens, vegetable soup	0	1 or 2	≥3
(4) Fruits	Fruits and fruit salad	0	1 or 2	≥3
(5) Dairy products	Milk, milk and coffee, cheese, yoghurt	0	1, 2, or ≥4	3
(6) Meat, fish, and eggs	Beef/poultry, fish/seafood, eggs	0	≥3	1 or 2
(7) Ultraprocessed sugary foods	Candies, chocolate bars, ice cream, with frosting/filling, sandwich cookies, breakfast cereal	≥ 2	1	0
(8) Sugary drinks	Soft drinks, fruit juices, chocolate milk	≥ 2	1	0
(9) Ultraprocessed savoury snacks and sausages	Savoury snacks (pizza/hamburger/hot dog), French fries, chips, instant noodles, sausages	≥ 2	1	0
(10) Water	Water	0	1, 2, 3, or 4	5 or 6

**Table 2:** Item discrimination and difficulty parameters and their respective standard errors estimated by item response theory analysis for the development of the Schoolchildren’s Diet Quality Scale (metric 0, 1).

Item	Parameter					
	*a*	SE (*a*)	*b*_1_	SE (*b*_1_)	*b*_2_	SE (*b*_2_)
1. Cereals, pasta, breads, roots, and tubers	0.68	0.11	−3.34	0.51	1.56	0.25
2. Beans	1.05	0.14	−0.32	0.09	−0.10	0.08
3. Vegetables and leafy greens	0.77	0.11	0.23	0.10	3.13	0.40
4. Fruits	0.75	0.10	−0.20	0.11	3.36	0.43
5. Dairy products	0.97	0.12	−1.11	0.14	2.24	0.25
6. Meat, fish, and eggs	0.75	0.11	−0.91	0.16	−0.35	0.11
7. Ultraprocessed sugary foods	0.81	0.11	−1.40	0.19	0.54	0.12
8. Sugary drinks	0.71	0.11	−0.27	0.12	1.86	0.26
9. Ultraprocessed savoury snacks and sausages	1.04	0.13	−0.90	0.12	0.55	0.10
10. Water	1.85	0.48	0.05	0.11	2.45	0.47

*a*, Discrimination parameter; *b*_1_, difficulty parameter in the intermediate category (category 1); *b*_2_, difficulty parameter in the higher category (category 2); SE, standard error.

**Table 3:** Distribution of items on the Schoolchildren’s Diet Quality Scale developed by item response theory analysis on the basis of response categories and their respective levels (metric 100, 10).

Levels of Diet Quality															
Very Poor					Poor		Reasonable		Good			Very Good			
70	75	80	85	90	95	100	105	110	115	120	125	130	135	140	145
C_1_					D_1_	B_1_	F_1_	V_1_	USF_2_		C_2_	D_2_		V_2_	F_2_
					M_1_		SD_1_		SSS_2_		SD_2_	W_2_			
					SSS_1_		W_1_								
					USF_1_		M_2_								
							B_2_								

C_1_, consumption of item 1 foods (cereals, pasta, breads, roots, and tubers) in the intermediate category (1–4 or ≥ 7 times/day). C_2_, consumption of item 1 foods in the higher category (5 to 6 times/day). B_1_, consumption of item 2 foods (beans) in the intermediate category (≥3 times/day). B_2_, consumption of item 2 foods in the higher category (1 to 2 times/day) V_1_, consumption of item 3 foods (vegetables and leafy greens) in the intermediate category (1 to 2 times/day). V_2_, consumption of item 3 foods in the higher category (≥3 times/day). F_1_, consumption of item 4 foods (fruits) in the intermediate category (1 to 2 times/day). F_2_, consumption of item 4 foods in the higher category (≥3 times/day). D_1_, consumption of item 5 foods (dairy products) in the intermediate category (1, 2, or ≥4 times/day). D_2_, consumption of item 5 foods in the higher category (3 times/day). M_1_, consumption of item 6 foods (meat, fish, and eggs) in the intermediate category (≥3 times/day). M_2_, consumption of item 6 foods in the higher category (1 to 2 times/day) USF_1_, consumption of item 7 foods (ultraprocessed sugary foods) in the intermediate category (1 time/day). USF_2_, consumption of item 7 foods in the higher category (0 times/day). SD_1_, consumption of item 8 beverages (sugary drinks) in the intermediate category (1 time/day). SD_2_, consumption of item 8 beverages in the higher category (0 times/day). SSS_1_, consumption of item 9 foods (ultraprocessed savoury snacks and sausages) in the intermediate category (1 time/day). SSS_2_, consumption of item 9 foods in the higher category (0 times/day).

**Table 4:** Description of the Schoolchildren’s Diet Quality Scale developed by item response theory analysis based on response categories ^a^.

Diet Quality	Description
Level 1: **Very Poor** ***θ* < 95**	Unhealthy foods are consumed ≥2 times/day, whereas healthy foods are not consumed (lower category), except cereals, pasta, breads, roots, and tubers 1–4 or ≥7 times/day (intermediate category)
Level 2: **Poor** **95** **≤ *θ* < 105**	Unhealthy foods are still consumed, but some at a lower frequency: ultraprocessed sugary foods and ultraprocessed savoury snacks and sausages are consumed 1 time/day (intermediate category). The consumption of cereals, pasta, breads, roots, and tubers remains in the intermediate category. Dairy products are consumed 1–2 or ≥4 times/day, and meat, fish, and eggs, ≥3 times/day (intermediate categories). Some schoolchildren consume beans ≥3 times/day (intermediate category)
Level 3: **Reasonable** **105** **≤ *θ* < 115**	The consumption of cereals, pasta, breads, roots, and tubers and dairy products still occurs at a frequency below or above the recommended (intermediate category). The consumption of other healthy foods begins in the intermediate category: fruits, 1–2 times/day and water, 1–4 times/day. Beans and meat, fish, and eggs are consumed 1–2 times/day (higher category). The consumption of sugary drinks is limited to 1 time/day (intermediate category), but that of ultraprocessed sugary foods and ultraprocessed savoury snacks and sausages remains in the intermediate category (1 time/day).
Level 4: **Good** **115** **≤ *θ* < 130**	All healthy foods are consumed at the higher category (beans and meat, fish, and eggs) or intermediate category (cereals, pasta, breads, roots, and tubers; dairy products; fruits; water; and vegetables and leafy greens). Unhealthy foods (ultraprocessed sugary foods and ultraprocessed savoury snacks and sausages) are no longer consumed, and some children do not consume sugary drinks.
Level 5: **Very Good** ***θ* ≥ 130**	Unhealthy foods (ultraprocessed sugary foods, ultraprocessed savoury snacks and sausages, and sugary drinks) are not consumed. The consumption of dairy products (3 times/day) and water (5–6 times/day) is increased to the higher category. Other healthy foods are consumed at the recommended frequency (higher category: cereals, pasta, breads, roots, and tubers; beans; and meat, fish, and eggs) or close to the recommended level (intermediate category: fruits and vegetables and leafy greens). Some children consume fruits and vegetables and leafy greens ≥3 times/day (higher category).

^a^ The lower category corresponds to the non-consumption of healthy food items and/or to the consumption of unhealthy food items ≥2 times/day; the intermediate category includes the consumption of healthy food items at a frequency below or above the DGBP recommendations and the consumption of unhealthy food items 1 time/day; and the higher category corresponds to the consumption of healthy and unhealthy food items at the recommended frequency according to the DGBP [25,26].

**Table 5:** Estimates (percentage and margin of error) of schoolchildren (*n* 6323) according to diet quality levels based on three Web-CAAFE survey years.

Survey Year	Schoolchildren’s Diet Quality Scale					p *
	Very Poor	Poor	Reasonable	Good	Very good	
2013 (*n* 1934)	20.8 (2.5)	40.2 (2.7)	28.8 (2.7)	9.8 (1.8)	0.4 (0.4)	0.2666
2014 (*n* 1980)	23.7 (2.9)	41.3 (3.5)	26.8 (2.5)	8.0 (1.8)	0.3 (0.2)	
2015 (*n* 2409)	20.9 (3.9)	38.1 (3.5)	30.9 (3.1)	9.6 (2.0)	0.4 (0.4)	
Family income (R$)						
1º tertile (*n* 2221)	19.2 (2.7)	40.7 (3.1)	30.0 (2.4)	9.9 (1.7)	0.2 (0.1)	0.1221
2º tertile (*n* 2067)	23.0 (3.5)	39.8 (3.5)	28.4 (2.9)	8.2 (1.6)	0.6 (0.6)	
3º tertile (*n* 2035)	24.3 (2.4)	38.4 (3.1)	27.6 (3.1)	9.3 (2.0)	0.3 (0.4)	

Percentage and margin of error considering sample weights. * The Rao-Scott chi-square test was used to analyse differences between survey years and between tertiles of family income.

## Supplementary Materials

The following are available online at https://www.mdpi.com/article/10.3390/nu13093175/s1, Figure S1: Scree plot of 10 items of Web-CAAFE (Food Intake and Physical Activities of Schoolchildren) from the full information factor analysis, Figure S2: Healthy foods item’s characteristic curves (The dotted line indicates the lower response category, the dashed line indicates the intermediate category, and the solid line indicates the higher category), Figure S3: Unhealthy foods item’s characteristic curves (The dotted line indicates the lower response category, the dashed line indicates the intermediate category, and the solid line indicates the higher category), Table S1: Factor loadings for each item generated by full-information factor analysis; Table S2: Characteristics of the representative sample analysed according to Schoolchildren’s Diet Quality Scale levels in surveys 2013–2015 (*n* 6,323).
