# Supplementary material for: Development of a Food-Based Diet Quality Scale for Brazilian Schoolchildren Using Item Response Theory
Source: Nutrients. 2021 Sep 12;13(9):3175. doi: 10.3390/nu13093175 (PMC8469312; doi:10.3390/nu13093175)
Supplement: Supplementary file 1 [file nutrients-13-03175-s001.zip › nutrients-1369423-supplementary.pdf]

Supplementary Materials

Table S1. Factor loadings for each item generated by full-information factor analysis.

**Item**	**Factor loadings**
1. Cereals, pasta, breads, roots, and tubers	0.37
2. Beans	0.53
3. Vegetables and leafy greens	0.41
4. Fruits	0.40
5. Dairy products	0.49
6. Meat, fish, and eggs	0.40
7. Ultraprocessed sugary foods	0.43
8. Sugary drinks	0.38
9. Ultraprocessed savoury snacks and sausages	0.52
10. Water	0.74
Percent of explained variation	28.0%

Table S2. Characteristics of the representative sample analysed according to Schoolchildren's Diet Quality Scale levels in surveys 2013-2015 (*n* 6,323).

**Characteristics**	**Survey year**			**Total**
	**2013 (*n* 1,934)**	**2014 (*n* 1,980)**	**2015 (*n* 2,409)**	**(*n* 6,323)**
	***n* (%)**	***n* (%)**	***n* (%)**	***n* (%)**
**Sex**				
Boys	989 (51.1)	982 (49.6)	1,230 (51.1)	3,201 (50.6)
Girls	945 (48.9)	998 (50.4)	1,179 (48.9)	3,122 (49.4)
**Age (years)** (Mean±SD)	9.6±1.2	9.5±1.1	9.7± 1.2	9.6±1.2
**Weight status**[a]				
Non overweight	1,474 (76.2)	1,432 (72.3)	1,779 (73.8)	4,685 (74.1)
Overweight (including obesity)	460 (23.8)	548 (27.7)	630 (26.2)	1,638 (25.9)
**Family income** (R$)[b] (Mean±SD)	2,015±1,060	2,053± for	2,046±996	2,039±1,001

[a]IOTF International Obesity Task Force [21]

[b]Monthly family income based on the school census sector [22]; 1 U$ = R$ 2,61 (average exchange rate between 2013 and 2015)

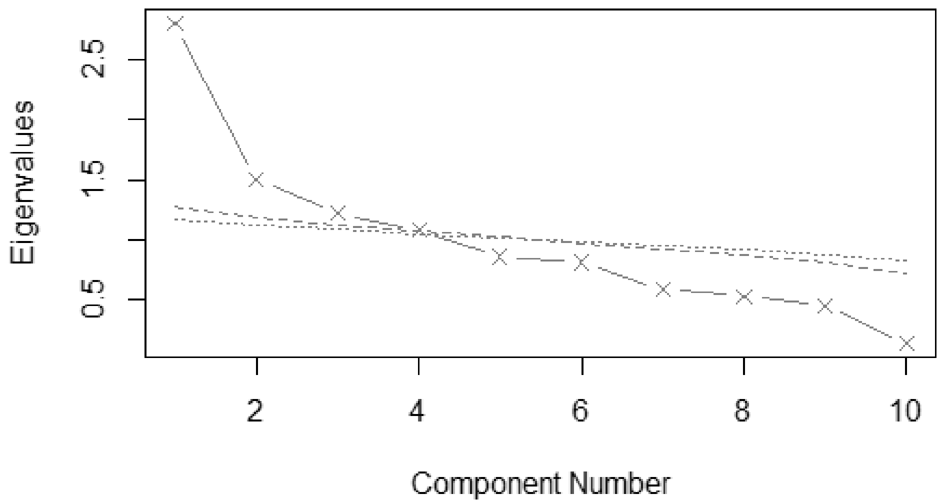

Figure S1. Scree plot of 10 items of Web-CAAFE (Food Intake and Physical Activities of Schoolchildren) from the full information factor analysis.

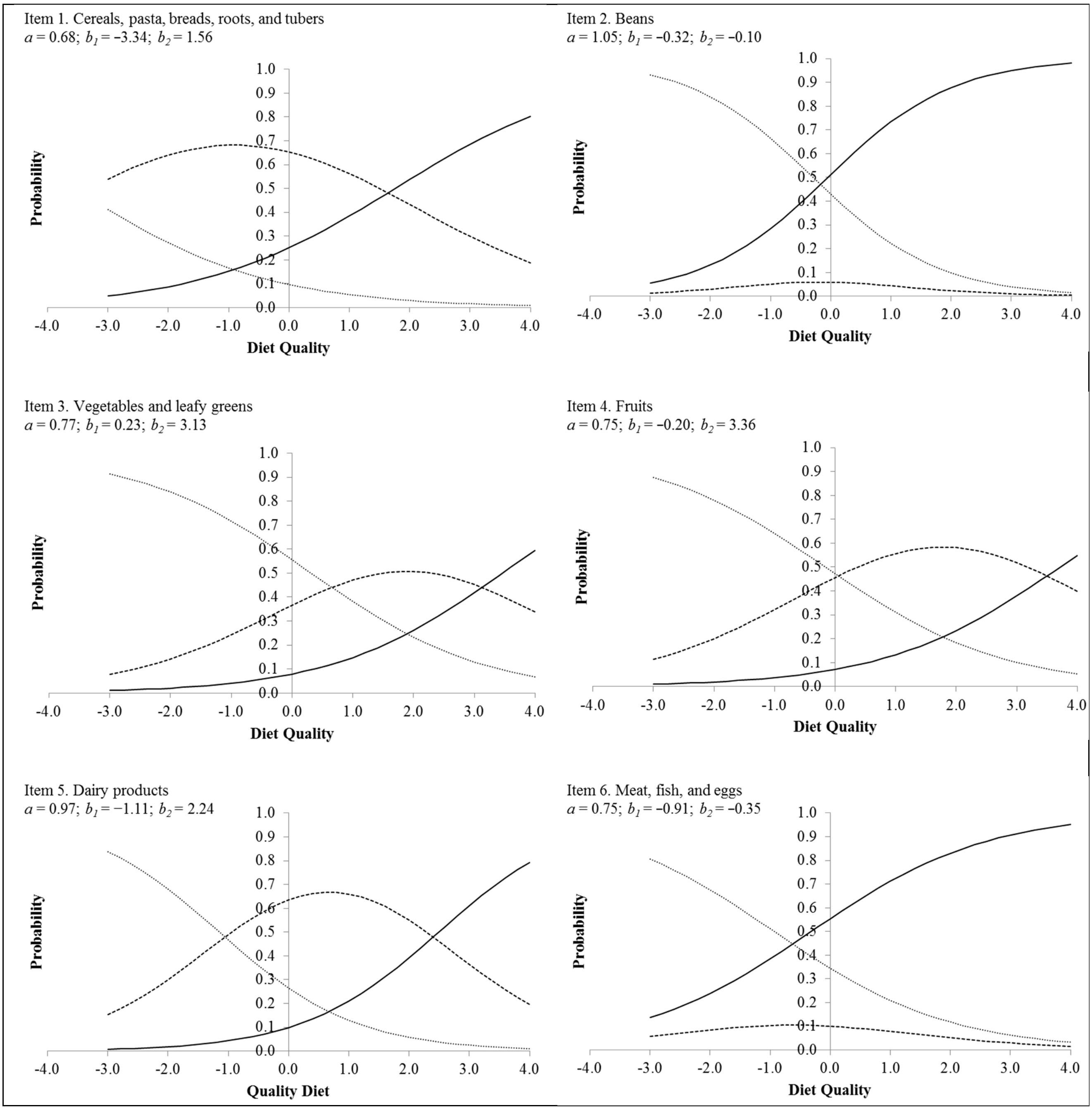

Figure S2. Healthy foods item's characteristic curves (The dotted line indicates the lower response category, the dashed line indicates the intermediate category, and the solid line indicates the higher category).

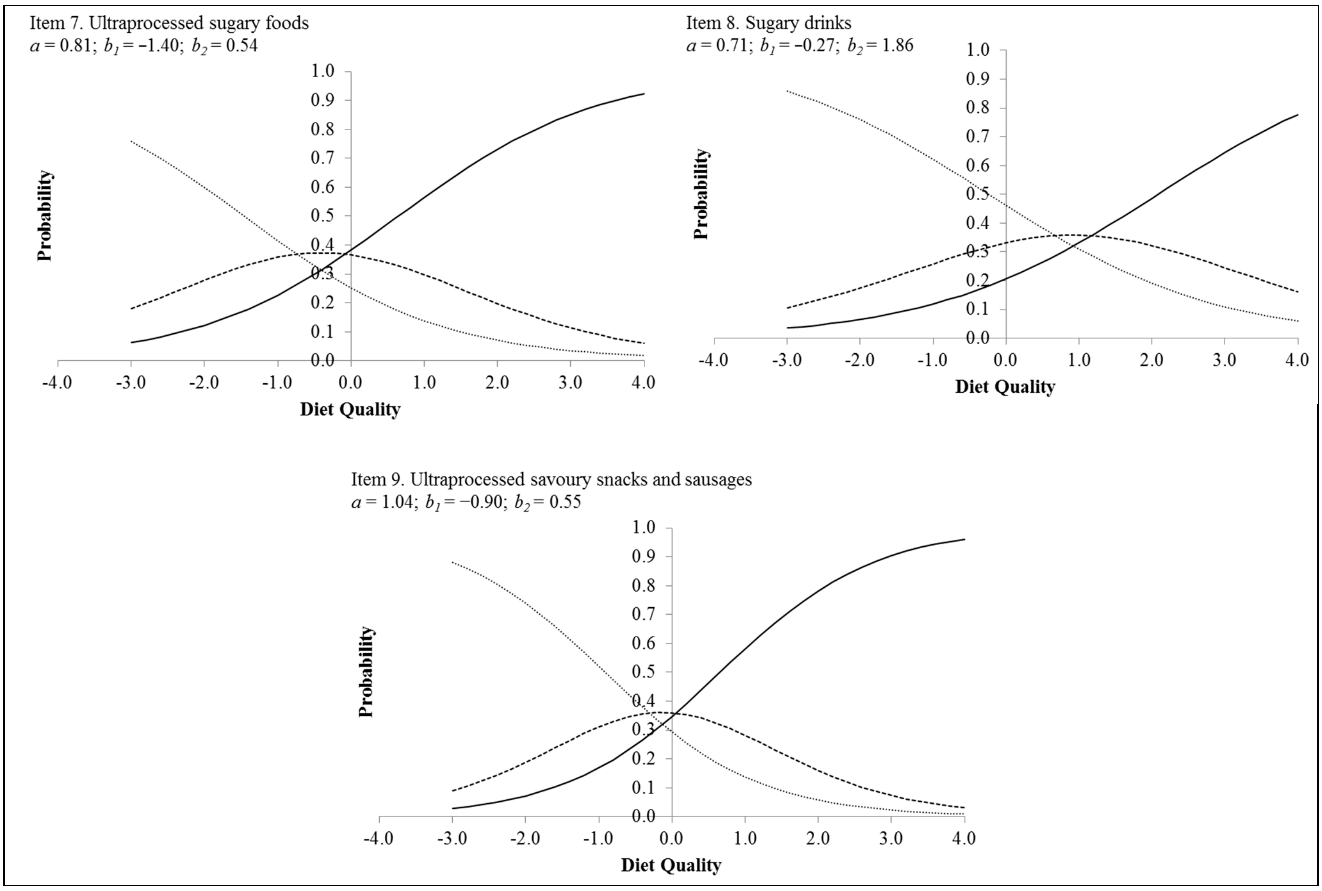

Figure S3. Unhealthy foods item's characteristic curves (The dotted line indicates the lower response category, the dashed line indicates the intermediate category, and the solid line indicates the higher category).
